# Reasons Low-Income Parents Offer Snacks to Children: How Feeding Rationale Influences Snack Frequency and Adherence to Dietary Recommendations

**DOI:** 10.3390/nu7075265

**Published:** 2015-07-21

**Authors:** Rachel E. Blaine, Jennifer Orlet Fisher, Elsie M. Taveras, Alan C. Geller, Eric B. Rimm, Thomas Land, Meghan Perkins, Kirsten K. Davison

**Affiliations:** 1 Department of Family and Consumer Sciences, California State University, Long Beach, 1250 Bellflower Blvd, FCS FA-15, Long Beach, CA 90840, USA; 2 Department of Nutrition, Harvard T.H. Chan School of Public Health, 665 Huntington Ave, Building 2, Boston, MA 02115, USA; E-Mails: erimm@hsph.harvard.edu (E.B.R.); kdavison@hsph.harvard.edu (K.K.D.); 3 Department of Public Health, Center for Obesity Research and Education, Temple University College of Public Health, 3223 N. Broad Street, Suite 175, Philadelphia, PA 19140, USA; E-Mail: jofisher@temple.edu; 4 Division of General Academic Pediatrics, Department of Pediatrics, MassGeneral Hospital for Children, 100 Cambridge Street, 15th floor, Boston, MA 02114, USA; E-Mails: elsie.taveras@mgh.harvard.edu (E.M.T.); meperkins@mgh.harvard.edu (M.P.); 5 Department of Social and Behavioral Sciences, Harvard T.H. Chan School of Public Health, Kresge 718, 677 Huntington Ave, Boston, MA 02115, USA; E-Mail: ageller@hsph.harvard.edu; 6 Office of Data Management and Outcomes Assessment, Massachusetts Department of Public Health, Boston, MA 02108, USA; E-Mail: thomas.land@state.ma.us

**Keywords:** snacks, parenting, childhood obesity prevention, child feeding

## Abstract

Although American children snack more than ever before, the parental role in promoting snacking is not well understood. In 2012–2013 at baseline in an intervention study to prevent childhood obesity in low-income Massachusetts communities, *n =* 271 parents of children aged 2–12 years completed surveys regarding nutritive and non-nutritive reasons they offered children snacks, demographics, and dietary factors. An analysis of variance demonstrated that parents reported offering snacks (mean/week; standard deviation (SD)) for nutritive reasons like promoting growth (x̄ = 2.5; SD 2.2) or satisfying hunger (x̄ = 2.4; SD 2.1) almost twice as often as non-nutritive reasons like keeping a child quiet (x̄ = 0.7; SD 1.5) or celebrating events/holidays (x̄ = 0.8; SD 1.1). Parents reported giving young children (2–5 years) more snacks to reward behavior (1.9 *vs.* 1.1, *p <* 0.001), keep quiet (1.0 *vs.* 0.5, *p <* 0.001), and celebrate achievements (1.7 *vs.* 1.0, *p <* 0.001) than parents of older children (6–12 years). Multivariable logistic regression models were used to obtain adjusted odds ratios, which indicated reduced child adherence to dietary recommendations when parents offered snacks to reward behavior (Odds Ratio (OR) = 0.83; 95% Confidence Interval (CI) 0.70–0.99), celebrate events/holidays (OR = 0.72; 95% CI 0.52–0.99), or achievements (OR = 0.82; 95% CI 0.68–0.98). Parental intentions around child snacking are likely important targets for obesity prevention efforts.

## 1. Introduction

In the United States children aged 2–12 years are consuming snack foods more frequently [1] and in greater quantities than ever before, eating an estimated 30% of daily calories in the form of sweet and salty snacks and up to 40% when sugar sweetened beverages are considered [2,3]. Snack foods tend to be low in fiber, vitamins, and minerals and high in refined flour, sodium, and sugar [4]. Greater snacking frequency has been associated with consumption of energy-dense foods (e.g., cookies, chips, sweets) and an increased risk for excessive weight gain in childhood [5,6,7,8]. Although large-scale public health efforts may be slowing the incidence of obesity in young children, nearly one in three American children are already overweight or obese by the time they begin elementary school [9,10]. Low-income Hispanic/Latino and African American children are disproportionately more likely than white children to experience obesity and its related complications [11,12].

Although children’s snacking habits are believed to be significant in the context of obesity risk, little is known about intentions of parents in promoting child snacking [8,13]. Additionally, the knowledge gap regarding parents’ snacking intentions is widest for families from low socioeconomic and racially/ethnically diverse backgrounds, where children are most likely to be overweight or obese [11,14,15,16]. The reasons parents offer snacks are important because they shape contexts in which children learn to eat. It has been hypothesized that non-nutritive feeding strategies which focus on outcomes unrelated to a child’s growth and hunger (e.g., behavior management, rewards) may lead to children having more frequent opportunities to eat in the absence of hunger, thus limiting their ability to successfully assess their own fullness [8]. Routine use of non-nutritive feeding practices has been associated with children’s increased energy intake, higher body mass index (BMI), and aversions to eating healthy foods [17,18,19]. Conversely, less is known about the influence of nutritive feeding purposes in which parents offer children snacks based on reasons which focus on a child’s health (e.g., satiate hunger, encourage growth).

We present baseline data collected as part of a community intervention to prevent and control obesity among multi-ethnic children aged 2–12 years from predominantly low-income communities in Massachusetts. Using self-reported survey data from a sample of low-income parents of children (*n =* 271), we present our findings based on the following research questions: (1) How frequently do parents offer snacks for nutritive and non-nutritive purposes? (2) What parent, child, and family-level characteristics are associated with low-income parents offering snacks to children for nutritive and non-nutritive purposes? (3) Are parent reasons for offering snacks associated with children’s adherence to obesity-related dietary recommendations? We hypothesized that parents who offered more snacks for non-nutritive reasons would be more likely to have children who fail to meet dietary recommendations related to obesity prevention. Understanding the motivation for and frequency with which parents offer snacks to children is essential to developing public health interventions that can address child snacking in the context of healthy eating.

## 2. Methods

### 2.1. Participants and Study Designs

This study utilizes cross-sectional baseline supplemental survey data collected between July 2012 and April 2013 from the Massachusetts Childhood Obesity Research Demonstration (MA-CORD) project, a two-year, multi-level, multi-sector community intervention to prevent and control obesity among children 2–12 years from predominantly low-income communities in Massachusetts. Detailed information about the larger study design and procedures have been published elsewhere [20,21]. Trained research assistants recruited parents onsite or by phone following a clinical visit at one of three community health centers (CHCs) in Massachusetts. Parents completed a survey on behalf of an index child and were eligible to participate if they met the following criteria: were at least 18 years old; had a child aged 2–12 years; spoke English, Spanish or Portuguese; resided in Fitchburg, New Bedford, or Lowell, Massachusetts; planned to stay at the CHC for the next two years. If parents had multiple age-eligible children, one child was randomly selected. If the index child had a serious nutrition or growth-related medical condition (e.g., cystic fibrosis, juvenile diabetes), the parent was excluded from the study. A response rate cannot be reported since child and parent-level data for all eligibility criteria were unavailable. Upon screening, 8% (*n =* 45) of parents were ineligible and fewer than 7% (*n =* 37) of 552 eligible parents declined to participate.

Research assistants administered survey questionnaires orally in English, Spanish or Portuguese to 515 parents. As a supplement to the main intervention survey assessing the intervention’s primary outcomes (*i.e.*, index child’s obesity-related behaviors and quality of life), parents were invited to participate in a supplemental survey that collected more specific information about child snacking, nutrition habits, and parent characteristics; 53% (*n =* 271) of parents opted-in. Parents also consented to allow survey data to be linked with weight and height data from their child’s electronic health record at the CHC. Participants received $15 for participation in both surveys. The study protocol was approved by the human subjects committees of the Massachusetts Department of Public Health, Harvard T.H. Chan School of Public Health, Massachusetts General Hospital, and Harvard Pilgrim Health Care Institute in June 2012 (#331765).

### 2.2. Measures

#### 2.2.1. Reasons Parents Offer Children Snacks

To assess parent reasons for giving children snacks, we used questions developed for this study by subject matter experts (Kirsten K. Davison, Jennifer Orlet Fisher) [22]. Parents indicated the frequency with which snacks were offered for a particular reason during a typical week. As no explicit definition of snacking was provided, it was left to parents to self-define what a snack meant to them, consistent with existing studies of child snacking [8]. Parents were prompted with the following: “These next questions are about snacks. People may think about snacks and snacking in very different ways. We would like to know more about what snacking means to you.” A total of six reasons were assessed, including two nutritive snack feeding reasons (“To help child grow”; “Because child is hungry”) and four non-nutritive reasons (“Reward for good behavior”; “To keep child quiet”; “To celebrate an event or holiday”; “To celebrate a child’s achievement”). Questions were coded based on frequency of snacks offered by reason (e.g., How often do you give your child snacks as a reward for good behavior? 0 = Never, 0.5 = Less than once per week, 1 = Once per week, 2 = Twice per week, 3 = Three times per week, 4 = Four times per week, 5 = Five or more times per week). See Supplementary Table S1 for complete questionnaire text used.

#### 2.2.2. Adherence to Childhood Dietary Recommendations

Participants reported the frequency in the past month with which their child consumed various foods (e.g., “In the past month, on average, how often did your child drink any regular (not diet) sodas or soft drinks, including Malta or Penafiel? Would you say never, less than once per week, One time per week, 2–4 times per week, Nearly daily/daily, 2–4 times per day, 5+ times per day?”) (Table S1). Questionnaire items measuring child dietary intake were previously validated for use in preschool-aged children regarding a child’s dietary intake of various food types during the previous month [23,24]. We found these items were also strongly positively correlated with the index child’s intake of the same foods and beverages on the previous day (Pearson ρ = 0.61). A binary indicator was used to assess child adherence (0 = No, 1 = Yes) to dietary recommendations for each of six possible healthy eating behaviors selected based on their relative contributions to dietary risk of childhood obesity [6,25].

Adherence to recommendations was determined using the following evidence-based cut-offs: (1) consumed soda less than 1 time per week [26]; (2) consumed sweetened juice drinks less than one time per week (punch, Kool-Aid^®^, Tampico, sports drinks) [26]; (3) limited 100% juice to one or fewer daily servings [27]; (4) consumed fast food less than one time per week [7] (5) consumed at least two servings/day of fresh, frozen or canned fruit; (6) consumed at least two servings/day of cooked/uncooked vegetables, excluding potatoes [6,7]. One point was assigned for each adherent behavior to develop a total score. The outcome of adherence was set at a score of 4 or greater out of six possible dietary behaviors, indicating that the child was engaging in a majority of adherent behaviors.

#### 2.2.3. Parent, Child, and Family Characteristics

Parents reported demographic information about themselves (relationship to child, gender, age, education, income, nativity status, language spoken, marital status), their child (age, gender, race/ethnicity), and their overall family (size of household, participation in assistance programs, level of food insecurity). Parent race/ethnicity was not assessed separately from their child. Household food security was assessed using a short-form of the United States Household Food Security Survey Module [28]. Child weight status was assessed using parent report of child gender and recent child weight and height measures obtained from electronic health records to obtain BMI-for-age growth percentiles using the 2000 Centers for Disease Control and Prevention cutoffs (e.g., overweight/obese: ≥85th percentile, normal: ≥5th and <85th percentile). Parent BMI (kg/m^2^) was obtained using self-reported current or pre-pregnancy weight and height.

### 2.3. Data Analysis

To describe participant characteristics we generated descriptive statistics including means, standard deviations, and frequency distributions. To assess differences in mean times per week that snacks were offered based on parent or child characteristics, we used one-way analysis of variance (ANOVA) and used least squares means to compare differences between racial/ethnic groups, using Scheffé’s method to adjust for multiple comparisons. We assessed adherence to dietary recommendations using multivariable logistic regression models including variables selected *a priori*, including child race, child age, child sex, child BMI *z*-score, parent BMI, missing parent BMI, and parent education, reporting odds ratios and 95% confidence intervals. We used SAS 9.3 (SAS Institute, Cary, NC, USA) to conduct all statistical analyses.

## 3. Results

### 3.1. Participant Characteristics

Demographic characteristics of parent participants (*n =* 271), their households, and their index child are presented in Table 1. The majority of the 271 participating parents were female, mothers, and primary caregivers to the child, with fewer fathers (9%), grandparents (3%), and legal guardians (1%) represented in the sample. Participant education was fairly evenly divided between not having completed high school, having a high school-level education, and having some college or technical school; few parents had completed college. Nearly half of parents were born outside the United States and spoke a language other than English. Among parents who provided self-reported weight and height, most parents were overweight or obese based on BMI. Approximately 10% of parents (*n =* 36) did not provide complete weight and height data needed to calculate BMI. Parents with missing BMI data were more likely to have lower household incomes, level of education, and children who were overweight or obese, though these differences were not statistically significant.

**Table 1:** Characteristics of parents of children aged 2–12 years in Massachusetts, USA (*n =* 271).

Parent Characteristics	*n*	(%)
**Relationship to child**		
Mother	235	86.7
Father	25	9.2
Grandparent	8	3.0
Legal guardian	3	1.1
**Gender**		
Female	245	90.4
Male	26	9.6
**Parent age in years (mean, range)**	32.1	(19–62)
**Parent education**		
Less than high school	75	27.7
High school graduate/GED	102	37.6
Some college or technical school	80	29.5
College graduate	14	5.2
**Parent birthplace**		
United States	151	55.7
Outside United States	120	44.3
**Parent language spoken**		
Only or mostly English	124	45.8
Equally English and another language	96	35.4
Only or mostly another language	51	18.8
**Parent marital status**		
Single	114	42.1
Married or living with partner	109	40.2
Separated/living apart from spouse	31	11.4
Divorced/widowed	17	6.3
**Parent body mass index**		
Normal	71	26.2
Overweight/obese	172	63.4
Missing	27	10.0
**Family characteristics**	*n*	(%)
**Household income**		
≤$10,000	85	31.3
$10,001 to $15,000	48	17.7
$15,001 to $20,000	54	19.9
$20,001 to $35,000	47	17.4
>$35,000	37	13.7
**Number in household (mean, range)**	4.0	(2–13)
**Family assistance received (select all that apply)**		
SNAP/EBT/food stamps	192	70.9
Free/reduced meals for child at school	188	69.4
WIC (Women Infants & Children)	103	38.0
**Family food insecurity in the past 12 months**		
Yes	146	53.9
No	125	46.1
**Child characteristics**	*n*	(%)
**Child age**		
Preschool-aged (2–5 years)	120	44.3
Elementary (6–10 years)	114	42.1
Middle (11–12 years)	37	13.6
**Child gender**		
Female	120	44.3
Male	151	55.7
**Child race**		
Hispanic/Latino	154	56.8
White	40	14.8
Black/African American	32	11.8
Multiracial	24	8.9
Other	21	7.7
**Child body mass index ^a^**		
Normal	152	56.1
Overweight	45	16.6
Obese	72	26.6
**Child adherence to dietary recommendations ^b^**	*n*	(%)
Soda (<1 time per week)	189	70.0
Sweetened juice drinks (<1 time per week)	80	29.5
100% juice (≤1 serving per day)	211	77.9
Fast food (<1 time per week)	173	63.8
Fruit (≥2 servings per day)	68	25.1
Vegetables (≥2 servings per day)	54	19.9

GED: General Educational Development exam for high school proficiency; SNAP: supplemental nutrition assistance program; EBT: electronic benefit transfer; ^a^ Using 2000 Center for Disease Control and Prevention (CDC) body mass index (BMI)-for-age growth percentiles calculated using parent report of child gender and weight/height measures obtained from child’s electronic medical record; ^b^ Based on parent self-report of child’s intake over previous month; ^c^ Assessed using the short form of the United States Household Food Security Survey Module.

Overall, the participant households were very low income, with 69% reporting combined earnings below the U.S. Census poverty threshold based on the median reported household size of four people [29]. Most parents received some type of food assistance from programs such as the Supplemental Nutrition Assistance Program (SNAP) or free/reduced meals for child at school and more than half reported that their household experienced food insecurity in the past year. More than half of children were identified by parents as being Hispanic/Latino.

A majority of children met dietary recommendations for consumption of soda, 100% fruit juice, and fast food; fewer children met guidelines for consumption of fruits and vegetables and limiting sweetened juice consumption. There was no significant difference in overall dietary overall adherence by child age (see Section 3.3), but preschool-aged children (2–5 years) were more likely than children six years or older to adhere to recommendations for soda (50% *vs.* 30%, *p* < 0.01), sweetened juice drinks (59% *vs.* 38%, *p* < 0.01), and fruit consumption (57% *vs.* 39%, *p* < 0.05).

### 3.2. Frequency of Parents Offering Snacks

Table 2 and Table 3 show differences in mean times per week parents offered snacks for different reasons (mean/week; standard deviation (SD)) across child characteristics (Table 2) and parent/family characteristics (Table 3). Overall, parents offered snacks for nutritive reasons more frequently in a given week than for non-nutritive reasons. Parents reported giving snacks to children to help them grow or to satisfy hunger almost twice as often as they did to keep a child quiet or celebrate an event or holiday.

**Figure 1:**
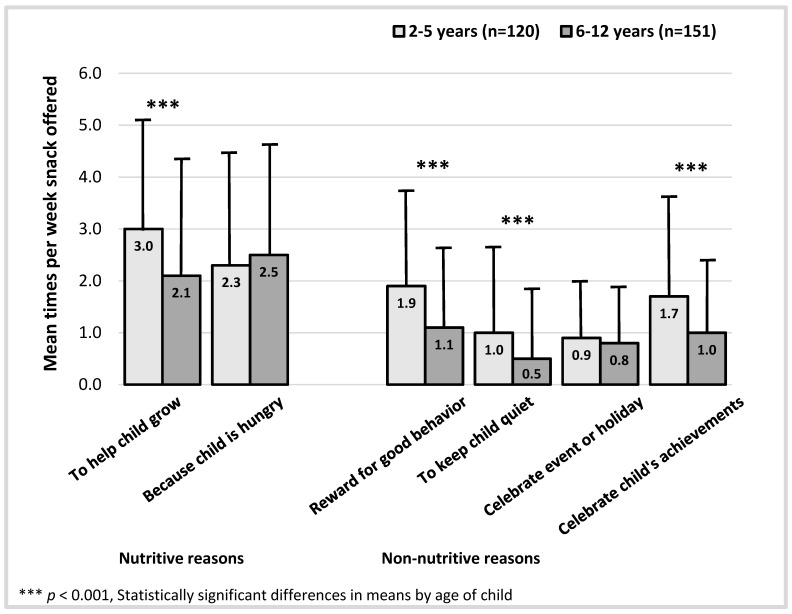
Differences in mean number of snacks offered per week by child age.

There were significant differences in the frequency with which parents provided snacks to children based on the age of the child (Figure 1). Notably, although parents of children aged 2–5 years offered more snacks to help their child grow when compared to parents of elementary-aged children aged 6–12 years, they also reported offering snacks for non-nutritive purposes almost twice as often to reward good behavior, keep a child quiet, and to celebrate a child’s achievements.

**Table 2:** Differences in mean times per week parent offered child snacks by demographic characteristics (*n =* 271)—child characteristics.

		Child BMI ^a^		Child Sex		Child Race/Ethnicity			
Times Per Week Parent Offered Snacks	Total (*n =* 271)	Normal (*n =* 152)	Overweight or Obese (*n =* 117)	Male (*n =* 120)	Female (*n =* 151)	Hispanic/Latino (*n =* 154)	Black/AA (*n =* 32)	White (*n =* 40)	Mixed Race (*n =* 24)
	Mean (SD)	Mean (SD)	Mean (SD)	Mean (SD)	Mean (SD)	Mean (SD)	Mean (SD)	Mean (SD)	Mean (SD)
**Nutritive reasons**									
To help child grow	2.5 (2.2)	2.7 (2.2)	2.2 (2.2)	2.8 (2.2)	2.3 (2.2)	2.4 (2.2)	**2.1 (2.2) ^b^**	2.9 (2.3)	**3.3 (2.0)**
Because child is hungry	2.4 (2.1)	2.5 (2.2)	2.2 (2.1)	2.5 (2.2)	2.3 (2.1)	**2.3 (2.2)^c^**	**1.5 (1.8)**	**3.5 (1.9) ^d^**	**2.2 (2.0)**
**Non-nutritive reasons**									
Reward for good behavior	1.5 (1.8)	1.5 (1.8)	1.4 (1.8)	1.6 (1.8)	1.4 (1.8)	1.5 (1.8)	1.0 (1.6)	1.3 (1.9)	1.3 (1.7)
To keep child quiet	0.7 (1.5)	0.7 (1.4)	0.8 (1.5)	0.7 (1.5)	0.8 (1.4)	0.8 (1.5)	0.5 (1.3)	0.3 (1.4)	0.6 (1.2)
Celebrate event or holiday	0.8 (1.1)	1.0 (1.3)	0.7 (0.9)	0.9 (1.2)	0.8 (1.0)	**0.9 (1.2) ^e^**	0.6 (0.9)	**0.5 (0.3)**	0.6 (0.4)
Celebrate child’s achievements	1.3 (1.7)	**1.6 (1.8)**	1.0 (1.4) **	1.5 (1.8)	1.2 (1.6)	**1.4 (1.7) ^e^**	1.3 (1.5)	**0.8 (1.2)**	0.9 (1.4)

BMI: Body mass index, AA: African American, SD: standard deviation; * *p <* 0.05, ** *p <* 0.01, ***p < 0.001; Statistically significant difference in means across characteristic; ^a^ Based on Centers for Disease Control and Prevention (CDC) body mass index (BMI)-for-age growth percentiles. Overweight/obese: ≥85th percentile, Normal: ≤5th and <85th percentile); ^b^ Significant difference compared with mixed race parents (*p <* 0.05); ^c^ Significant difference compared with black/African American (*p <* 0.05); ^d^ Significant difference compared with Hispanic/Latino (*p <* 0.001), Black/African American (*p <* 0.001), and mixed race (*p <* 0.05); ^e^ Significant difference compared with white (*p <* 0.05).

**Table 3:** Differences in mean times per week parent offered child snacks by demographic characteristics (*n =* 271)—parent and family characteristics.

			Parent Education		Parent BMI			Family Food Insecurity ^a^	
		Total (*n =* 271)	High School Graduate or Less (*n =* 177)	College or Technical School (*n =* 94)	Normal (*n =* 71)	Overweight (*n =* 77)	Obese (*n =* 122)	Yes (*n =* 146)	No (*n =* 125)
Times per week parent offered snacks		Mean (SD)	Mean (SD)	Mean (SD)	Mean (SD)	Mean (SD)	Mean (SD)	Mean (SD)	Mean (SD)
**Nutritive reasons**									
To help child grow		2.5 (2.2)	2.3 (2.2)	2.8 (2.2)	**2.9 (2.2) ^b^**	2.5 (2.1)	**2.2 (2.1)**	2.3 (2.2)	2.7 (2.2)
Because child is hungry		2.4 (2.1)	2.2 (2.1)	2.7 (2.2)	2.5 (2.1)	2.4 (2.2)	2.3 (2.1)	2.4 (2.2)	2.4 (2.1)
**Non-nutritive reasons**									
Reward for good behavior		1.5 (1.8)	**1.7 (1.9)**	1.0 (1.5) *	1.7 (1.9)	1.5 (1.8)	1.3 (1.7)	1.5 (1.8)	1.4 (1.8)
To keep child quiet		0.7 (1.5)	0.8 (1.5)	0.6 (1.4)	0.9 (1.7)	0.7(1.4)	0.7 (1.4)	0.8 (1.6)	0.7 (1.4)
Celebrate event or holiday		0.8 (1.1)	0.9 (1.2)	0.7 (1.0)	0.9 (1.2)	0.8 (1.0)	0.8 (1.1)	0.9 (1.2)	0.8 (1.0)
Celebrate child’s achievements		1.3 (1.7)	1.5 (1.7)	1.1 (1.6)	**1.7 (1.9) ^b^**	1.4 (1.8)	**1.1 (1.5)**	1.4 (1.7)	1.3 (1.7)

BMI: Body mass index, SD: standard deviation; * *p <* 0.05, ** *p <* 0.01, *** *p <* 0.001; Statistically significant difference in means across characteristic; ^a^ Using short form of United States Household Food Security Survey Module; ^b^ Significant difference compared with obese parents (*p <* 0.05).

Parents reported offering fewer snacks to overweight or obese children across both nutritive and non-nutritive reasons, though the difference was only statistically significant for snacks provided to celebrate a child’s achievements (1.0; SD 1.6 *vs.* 1.6; SD 1.8, *p <* 0.01). A few differences in snack feeding emerged across child race/ethnicity; white children received significantly more snacks because they were hungry when compared with Hispanic/Latino, black, and mixed race children. Across all racial/ethnic groups, black children received the fewest snacks for nutritive purposes. Hispanic children received snacks more frequently for non-nutritive purposes than children of other racial backgrounds, especially for reasons related to celebration. White children received the fewest number of snacks for non-nutritive reasons.

When compared with college-educated parents, those with a high school diploma or less were more likely to give snacks for non-nutritive reasons and less likely for nutritive reasons. Overall, obese parents reported offering their children fewer snacks for both nutritive and non-nutritive purposes when compared to normal weight parents, offering significantly fewer snacks to help children grow and to celebrate a child’s achievements. Family food insecurity was not significantly associated with any differences in snack frequency.

### 3.3. Snack Offerings and Child Adherence to Dietary Recommendations

We compared the frequency with which parents offered snacks for different reasons with the likelihood that their child adhered to current dietary recommendations related to obesity prevention, defined as meeting a majority of possible food and beverage recommendations in the previous month (Table 4).

**Table 4:** Association between reasons low-income parents offer snacks to children and child adherence to dietary recommendations in previous month (*n* = 271).

Times Per Week Parent Offered Snack to Child ^b^	Referent Group: Child Adhering to the Majority of Dietary Recommendations in the Previous Month (*n* = 81) ^a^
	OR (95% CI) ^c^
**Nutritive reasons**	
To help child grow	1.05 (0.92, 1.19)
Because child is hungry	0.88 (0.77, 1.01)
**Non-nutritive reasons**	
Reward for good behavior	0.83 (0.70, 0.99) *
To keep child quiet	0.89 (0.73, 1.08)
Celebrate event or holiday	0.72 (0.52, 0.99) *
Celebrate child’s achievements	0.82 (0.68, 0.98) *

* *p* < 0.05, Statistically significant odds ratios; ^a^ Met at least four of six recommendations for recent dietary intake: Over past month (1) consumed soda <1 time per week; (2) consumed sweetened juice drinks <1 time per week (punch, Kool-Aid^®^, Tampico, sports drinks); (3) limited 100% juice to ≤1 daily serving; (4) consumed fast food <1 time per week (5) consumed 2+ servings/day of fresh, frozen or canned fruit; (6) consumed 2+ servings/day of cooked/uncooked vegetables, excluding potatoes; ^b^ Measured in times per week parent offers child snacks; ^c^ Odds ratio adjusted for child race, child age, child sex, child BMI z-score, parent BMI, missing parent BMI, and parent education using logistic regression.

Overall, parents who offered snacks more frequently per week for non-nutritive reasons reported child dietary intake patterns that reflected lower adherence to dietary recommendations. Children whose parents offered snacks more frequently per week to reward good behavior (OR 0.83; CI 0.70–0.99), celebrate an event or holiday (OR 0.72; CI 0.52–0.99), or celebrate a child’s achievements (OR 0.82; CI 0.68–0.98) were significantly less likely to adhere to recommendations (*p <* 0.05). There were no significant associations between nutritive snack feeding reasons and adherence to dietary recommendations.

## 4. Discussion

This is the first study of its kind to describe low-income parents’ frequency and rationales for offering their children snacks, as well as the association between parent reasons for providing snacks and child adherence to dietary recommendations. We found that parents reported offering their children snacks for a variety of both nutritive and non-nutritive reasons that differed by child age, weight status, and race/ethnicity. The reasons parents report offering snacks may influence the likelihood of children adhering to dietary recommendations. Children of parents who reported offering non-nutritive snacks more frequently were less likely to adhere to current dietary recommendations related to obesity prevention than parents who didn’t report offering snacks for these reasons.

One encouraging finding of our study is that low-income parents reported offering snacks for nutritive reasons (e.g., because child is hungry) more frequently in a usual week than non-nutritive reasons. A recent qualitative study of low-income white, Hispanic, and African American parents found that parents who described their preschooler’s hunger as an important reason for offering snack foods also described offering more healthy foods to their children [30]. We also found that a majority of parents reported that their child’s diet met recommendations for consumption of fast food, soda, and juice in the previous month (e.g., limited consumption to less than once per day or week). Parent education should build upon these practices by emphasizing the benefits of low-cost, healthy, nutrient-dense snacks like fruits, vegetables, whole grains, and lean proteins.

We found that parents reported offering snacks to preschool-aged children (2–5 years) at a significantly greater frequency than older children (6–12 years). In some respects, these findings are expected and even appropriate. To prevent obesity, the American Academy of Pediatrics recommends structured “healthy and nutritious” snacking, as opposed to ongoing grazing, suggesting that elementary-aged children have 1–2 snacks daily and toddlers up to three snacks [6]. Therefore, younger children may be more likely to consume a greater number of snacks in a given week simply due to their dietary needs perceived by parents. However, parents reported offering numerous snacks to young children for non-nutritive reasons (e.g., to reward behavior, keep child quiet, celebrate an accomplishment). Occasional rewards or celebrations may not be cause for concern, but cumulative opportunities to snack in a given week (e.g., reward for potty training, distraction to sit quietly through church, birthday party, holiday celebration) may cue young children to eat regardless of hunger. Exposure to healthy foods in early childhood is critical because children’s taste preferences may be established prior to entering elementary school [31] and using unhealthy foods to reward behavior has been shown to increase children’s preferences for such foods [32]. Parents of older children may also benefit from reminders about the importance of focusing on their children’s growth and hunger as a primary purpose in offering snacks, especially in light of the fact that consumption of sugary drinks nearly doubles once children reach elementary school [2].

Parents of black children reported offering fewer snacks for nutritive purposes and parents of Hispanic/Latino children offered a greater number of snacks for non-nutritive purposes when compared with white children. Our findings are consistent with other literature that has examined feeding intentions among low-income mothers of color. In one small qualitative study of low-income African American mothers of preschoolers, a participant explained that, “Snacks are not food,” describing the general consensus that snacks were important tools to manage a child’s behavior and did not require as much consideration for nutrient content compared with meals [33]. In addition to black parents, our sample represented a substantial proportion of Hispanic/Latino parents who were born outside of the United States (44%). A study of immigrant Latina mothers found that many reported offering more foods and snacks to their children upon arriving to the United States, especially fast food [34]. Findings from another study of low-income parents of preschoolers, 20% of whom were Hispanic/Latino immigrants, found that fast food items, even entire meals (e.g., Happy Meal^®^ including burger, fries, and drink) were categorized by some participants as “snacks” rather than meals. Half of the participants agreed that pizza was not a meal food [35]. Consequently, when the frequency of snacking is assessed it is important to also consider the content of these snacks.

We found that increases in snacking opportunities, specifically snacks offered for non-nutritive purposes, may reduce adherence to dietary recommendations which reduce the risk of childhood obesity, including limiting consumption of caloric beverages (e.g., soda, juice drinks, juice) and fast food, and consuming fruits and non-starchy vegetables. Perhaps parents who offer children snacks for non-nutritive reasons are more likely to use appealing foods that have more currency with respect to behavior modification (e.g., cake *versus* fruit), or that increased eating opportunities are paired with caloric beverages. To combat the rising rates of childhood obesity, parents of children of all ages should be encouraged to consider snack times as important opportunities to nourish children and limit “empty” calorie foods which are nutrient poor. Snacks with higher glycemic loads, such as sugary drinks, potato chips or candy, may contribute to increased cravings for more food and reduced satiety, with a possible consequence of overeating at future eating occasions [36,37,38]. A study of 115 elementary-school girls found that when snacks were offered ad libitum, girls offered lower glycemic snacks of cheese and vegetables consumed 72% fewer calories than girls offered potato chips [39]. Food insecure families may also be more likely to allow children unrestricted access to such snack foods when they are available, thus contributing to the observation of the “food insecurity-obesity paradox” [30,40,41].

Our study has limitations. Parents were asked about frequency of offering snacks to their children using items developed for this study, as a validated assessment tool was not available at the time. Since snacks were self-defined by parents, it is possible that variations in interpretation of the word “snack” may have influenced the observed outcomes. Additionally, our measurement of snacks came only from one parent’s perspective, and did not include other snacks a child might receive in school, day care, or from other family members. Desirability bias is also a possibility, as parents may be reluctant to quantify the frequency with which they provide snacks for socially undesirable reasons (e.g., to keep a child quiet), although the absolute frequency of snacks reported in our study appears to reflect national estimates for child snacking [3]. Forthcoming work should include the use of validated measures to assess parents’ snack feeding behaviors, including the specific quantity, quality, and context in which child snacking is occurring. Such measures could also be triangulated with parent food diaries or shopping receipts in order to determine which snacks are offered for nutritive *versus* non-nutritive purposes [42,43]. Since this was a cross-sectional analysis, reverse causation may exist with respect to child or parent weight status, as parents may already be restricting a child’s snack intake or adjusting their own behaviors in response to a perceived weight problem. Therefore, trends towards less reported snacks offered to overweight or obese children may not actually reflect the anticipated positive association, a phenomenon that has been described elsewhere in child snacking literature [8]. As this study only assessed snacks provided by one parent, we are unable to determine if overweight or obese children consumed more snacks when they were available in other contexts like school, day care, convenience stores, or at the homes of extended family members. Additionally, race/ethnicity was only collected for the child of interest, rather than the parent, so we are unable to identify differences in feeding practices by parent race/ethnicity. Nevertheless, this study is the first of its kind to describe multi-ethnic low-income parents’ motivations for offering snacks to their children. 

## 5. Conclusions

Parent reasons for feeding children snacks may influence both the frequency of eating opportunities as well as the quality of a child’s diet. Future investigations should assess the longitudinal influence of parent snack feeding rationales on changes in children’s diet quality, food preferences, and body mass index. Validated measures of parent-derived definitions of snacks should be used, taking care to ensure tools capture nuanced interpretations that may differ by parent race/ethnicity, education, and income status. Early childhood obesity prevention efforts must consider the role of parents in promoting child snacking and provide capacity building around parenting strategies that utilize non-food rewards. Comprehensive approaches to obesity prevention should also address parents’ snack feeding strategies while working to improve environmental barriers to offering healthy snacks such as food security, access, and availability.

## Supplementary Files

Supplementary File 1
