# Supplementary material for: Reasons Low-Income Parents Offer Snacks to Children: How Feeding Rationale Influences Snack Frequency and Adherence to Dietary Recommendations
Source: Nutrients. 2015 Jul 21;7(7):5982–99. doi: 10.3390/nu7075265 (PMC4517042; doi:10.3390/nu7075265)
Supplement: Supplementary File 1 [file nutrients-07-05265-s001.docx]

Supplementary Materials

**Table S1.** Questionnaire used in this study.

| **Questionnaire Items Used to Assess Child Snacking** | **Response Options and Coding** |
| --- | --- |
| How often do you give your child snacks to help him or her grow?  How often do you give your child snacks because he or she is hungry?  How often do you give your child snacks as a reward for good behavior?  How often do you give your child snacks to keep him or her quiet?  How often do you give your child snacks to celebrate an event or holiday?  How often do you give your child snacks to celebrate your child’s achievements? | 0 = Never  0.5 = Less than once per week  1 = Once per week  2 = Twice per week  3 = Three times per week  4 = Four times per week  5 = Five or more times per week |
| **Questionnaire Items Used to Assess Child Diet Quality** | **Response Options and Coding** |
| In the past month, on average, how often did your child drink any regular (not diet) sodas or soft drinks, including Malta or Penafiel? (0 = Never, 1 ≤ 1/week,  2 = 1/week, 3 = 2–4 times/week, 4 = Nearly daily/daily, 5 = 2–4 times/day,  6 = 5+ times/day)  In the past month, on average, how often did your child drink any punch, Kool-Aid^®^, Tampico, sports drinks, Goya juice or other fruit-flavored drinks, not including  fruit juice?  In the past month, on average, how often did your child drink fruit juice?  Fruit juice is a drink, which is 100% juice, like orange juice, apple juice, or grape juice. Do not count punch, Kool-Aid^®^, Tampico, sports drinks, Goya juice, or other fruit-flavored drinks.  In the past month, on average, how often did your child eat any vegetables? Please include all cooked and uncooked vegetables or salads. Do not count French fries, fried potatoes, or potato chips.  In the past month, on average, how often did your child eat fruit? Please think about all forms of fruits, including cooked or raw, fresh, frozen or canned. Do not count fruit juice.  In the past month, on average, how often did your child eat something from a fast food restaurant such as McDonald’s, Burger King, Taco Bell, Dunkin Donuts or a pizza place? Would you say … (0 = Never, 1 ≤ 1/week, 2 = 1/week,  3 = 2–4 times/week, 4 = Nearly daily/daily, 5 = 2–4 times/day, 6 = 5+ times/day) | 0 = Never  1 ≤ 1/week  2 = 1/week,  3 = 2–4 times/week,  4 = Nearly daily/daily,  5 = 2–4 times/day  6 = 5+ times/day) |
